# Peripheral “Swiss Cheese” Appearance in a COVID-19 Patient with Chronic Obstructive Pulmonary Disease

**DOI:** 10.4269/ajtmh.20-0605

**Published:** 2020-06-12

**Authors:** Maki Miwa, Mikio Nakajima, Hideaki Goto

**Affiliations:** Emergency and Critical Care Center, Tokyo Metropolitan Hiroo Hospital, Tokyo, Japan

**Figure 1.**
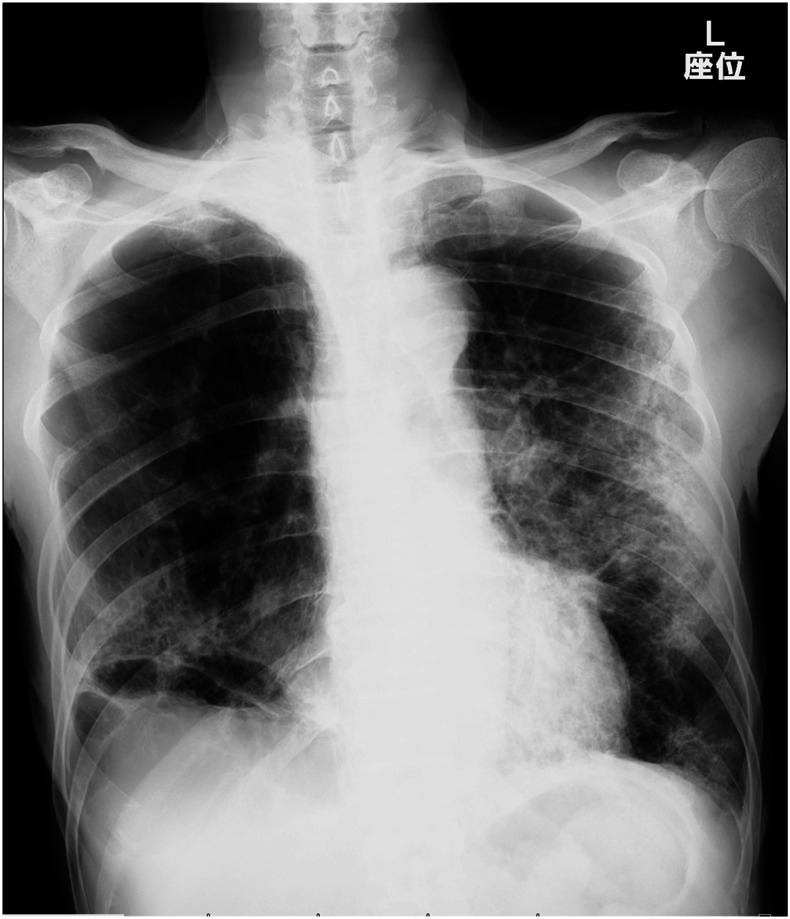
Chest X-ray. A chest X-ray revealed bilateral opacities peripherally.

**Figure 2.**
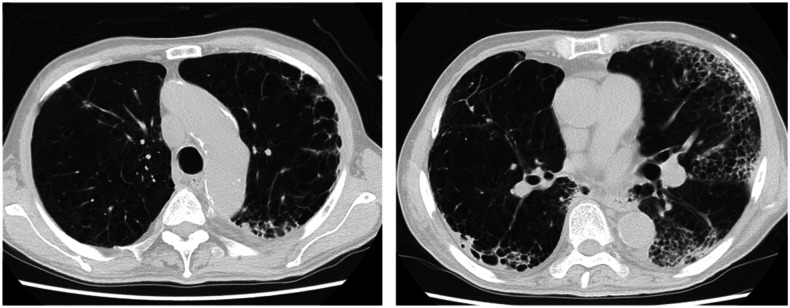
Computed tomography (CT) of the lung. Chest CT revealed diffuse low attenuation areas and increased concentrations along the circumference of the emphysema in a peripheral, bilateral, posterior, and lower lung zone distribution.

A 78-year-old Japanese man with a history of right upper lobectomy due to lung abscess was transferred to our emergency department for dyspnea. The patient experienced progressive episodes of exertional dyspnea over a two-year period because of underlying chronic obstructive pulmonary disease (COPD), which was unmanaged. The patient was a former smoker. He smoked 40 cigarettes per day for 28 years. On the day before admission, he experienced a significantly worse episode of dyspnea triggering his decision to visit a local clinic. On arrival, he presented with the following: body temperature, 37.3°C; respiratory rate, 30/minute; and oxygen saturation, 74% (room air). A chest X-ray (Figure 1) revealed bilateral opacities peripherally. Chest computed tomography (CT) (Figure 2) revealed diffuse low attenuation areas and increased concentrations along the circumference of the emphysema. Three weeks before admission, his wife was hospitalized because of COVID-19. A reverse transcription–polymerase chain reaction test for SARS-CoV-2 was positive.

Chest CT findings related to COVID-19 typically present with ground-glass opacities with or without consolidation in a peripheral, bilateral, posterior, and diffuse or lower lung zone distribution. Ground-glass opacities have also been reported to have round morphology or a “crazy paving” pattern.^1^ However, the combination of COVID-19 pneumonia and advanced structural lung damage caused by COPD can culminate in atypical CT findings such as a peripheral “Swiss cheese” appearance.^2^ The peripheral distribution of “Swiss cheese” appearances may denote COVID-19 pneumonia in patients with underlying COPD and could be exploited for use in its diagnosis.
